# S43. A PROOF-OF-MECHANISM STUDY OF THE PDE10 INHIBITOR RG7203 IN PATIENTS WITH PROBING REWARD FUNCTIONS WITH IMAGING AND BEHAVIORAL APPROACHES

**DOI:** 10.1093/schbul/sby018.830

**Published:** 2018-04-01

**Authors:** Daniel Umbricht, Jürgen Dukart, Markus Abt, Paul Tamburri, Chris Chatham, Michael Frank, Anne Collins, David Walling, Rick Mofsen, Daniel Gruener, Lev Gertsik, Jeffrey Sevigny

**Affiliations:** 1 F. Hoffman - La Roche Ltd; 2 Brown University; 3 CNS Network, LLC; 4 St. Louis Clinical Trials; 5 CCT

## Abstract

**Background:**

The enzyme phosphodiesterase 10A (PDE10A) is highly expressed in the striatum where it modulates both dopamine D2 and D1 dependent signaling. Its inhibition leads to a suppression of D2 mediated signaling –similar to effects of D2 antagonists - and an enhancement of D1 dependent signaling. D1-dependent signaling has been implicated in reward based learning. Its deficient activation may be a key factor underlying deficient reward functions including reward anticipation and reward based learning that have been implicated as major drivers of negative symptoms of schizophrenia. Therefore, inhibition of PDE10 could be a way to ameliorate such deficits and consequently negative symptoms. In healthy volunteers the PDE10 inhibitor RG7203 indeed enhanced performance in tasks that probed reward functioning suggestive of its potential utility to treat negative symptoms in schizophrenia. We therefore tested the hypothesis that it should enhance imaging and behavioral markers of reward functions in patients with moderate negative symptoms in order to establish mechanistic proof of its utility as treatment of negative symptoms.

**Methods:**

In a three-way cross-over study we investigated the effects of two doses of RG7203 (5 mg and 15 mg) and placebo given as adjunctive treatment to stable background antipsychotic treatment on reward functioning and reward-based effortful behavior using the monetary incentive delay (MID) task during fMRI and the effort choice task in patients with chronic schizophrenia and moderate levels of negative symptoms (PANSS negative symptom factor score ≥ 18 points). Each treatment period lasted three weeks followed by a 2 week washout period. fMRI and behavioral tasks were administered at the end of each treatment period. Key outcome measures were the differential BOLD during reward anticipation and overall BOLD activity during the MID task and the percentage of high-effort high-reward choices when the probability of reward was 100% during the effort choice task.

**Results:**

Thirty-three patients with schizophrenia (30 male; 21 B, 9 W, 3 A; mean age 36.6 ± 7 y; PANSS NSFS = 22.8 (±1.4) at screening) were recruited at three study centers in the US. Twenty-four subjects finished the entire study. RG7203 at 5 mg significantly increased differential BOLD activity during reward anticipation in the MID task. However, this enhancement occurred in the context of a significant decrease of BOLD activity across all conditions during the MID task under treatment with RG7203. RG7203 significantly worsened reward-based effortful behavior in the effort choice task (the high-effort high-reward choice: 67% for both doses of RG7203 versus 73% for placebo). Multiple regression revealed that the decrease in effortful behavior was significantly related to the decrease in overall BOLD activity during the MID task and not related to the difference of BOLD activity during reward anticipation versus the control condition.

**Discussion:**

In contrast to our expectation and previous results in healthy volunteers, RG7203 worsened indices of reward functions which we hypothesize may be due to a further enhancement of D2 antagonistic activity. The results do not support the utility of a PDE10 inhibitor as adjunctive treatment for negative symptoms in patients with schizophrenia. Given the previous observation that RG7203 enhanced reward functions in healthy volunteers who were not treated with D2 antagonist, the results of our study point to potentially deleterious effects of D2 blockade on reward functions and by extension on negative symptoms of schizophrenia. They raise the question if the presence of D2 antagonistic treatment curtails the potential effects of any adjunctive treatment for negative symptoms.

